# Admission Endothelial Activation and Stress Index and Echocardiographic RV-PA Coupling for Early Risk Stratification in Intermediate-Risk Acute Pulmonary Embolism

**DOI:** 10.3390/jcm15145675

**Published:** 2026-07-20

**Authors:** Fikret Keles, Alp Yildirim, Muzeyyen Gizem Parmak, Ahmet Ridvan Bilgic, Muhammet Salih Ateş, Erdoğan Sökmen

**Affiliations:** 1 Department of Cardiology, Faculty of Medicine, Kirsehir Ahi Evran University Training and Research Hospital, Kirsehir 40100, Türkiye; alp.yildirim@ahievran.edu.tr (A.Y.); m.gizem.parmak@ahievran.edu.tr (M.G.P.); muhammet.ates@ahievran.edu.tr (M.S.A.); erdoganmen@ahievran.edu.tr (E.S.); 2 Department of Chest Diseases, Kirsehir Ahi Evran University Training and Research Hospital, Kirsehir 40100, Türkiye; dr.ridvanbilgic@gmail.com

**Keywords:** pulmonary embolism, EASIX, endothelial activation and stress index, TAPSE/PASP, RV-PA coupling, lactate, troponin, risk stratification

## Abstract

**Background:** Intermediate-risk acute pulmonary embolism (PE) is clinically heterogeneous, and early deterioration may occur despite initial normotension. The Endothelial Activation and Stress Index (EASIX) is a readily available laboratory index reflecting endothelial activation, cellular injury, renal-perfusion stress, and platelet-related thromboinflammatory burden. We investigated whether admission EASIX and echocardiographic right ventricular-pulmonary arterial (RV-PA) coupling assessed by the TAPSE/PASP ratio identify intermediate-risk PE patients at increased risk for an early adverse clinical outcome (EACO). **Methods:** This retrospective cohort study included 900 consecutive intermediate-risk acute PE patients admitted between 1 January 2020 and 10 June 2025. EASIX was calculated as LDH (U/L) × creatinine (mg/dL)/platelet count (10^9^/L). The primary endpoint was EACO within the first seven days after PE diagnosis during the index hospitalization, including hemodynamic decompensation, vasopressor/inotrope requirement, intensive care unit transfer, rescue reperfusion therapy, ventilatory support, cardiac arrest, or PE-related death. Hierarchical logistic regression, ROC analysis, calibration assessment, bootstrap internal validation, and 10-fold cross-validation were performed. **Results:** EACO occurred in 110 patients (12.2%). Patients with EACO had higher EASIX (2.28 [1.52–3.27] vs. 1.27 [0.86–2.00], *p* < 0.001) and lower TAPSE/PASP ratio (0.29 [0.23–0.36] vs. 0.42 [0.33–0.57], *p* < 0.001). log2-EASIX correlated inversely with TAPSE/PASP (Spearman rho = −0.30, *p* < 0.001). In the final combined model, log2-EASIX (OR 1.55 per doubling, 95% CI 1.14–2.10, *p* = 0.005) and TAPSE/PASP per 0.1-unit decrease (OR 1.36, 95% CI 1.10–1.68, *p* = 0.004) remained independently associated with EACO after adjustment for clinical variables, troponin, lactate, D-dimer, and C-reactive protein-to-albumin ratio. The final model showed higher discrimination than the clinical-laboratory model, although the absolute AUC increment was modest (AUC 0.920 vs. 0.904). Bootstrap optimism-corrected AUC was 0.910 and 10-fold cross-validated AUC was 0.904. **Conclusions:** Admission EASIX and impaired RV-PA coupling may provide complementary prognostic information for early risk stratification in intermediate-risk acute PE. Because this was a retrospective single-center study without external validation, these findings should be considered hypothesis-generating and should not be used as definitive treatment-escalation triggers before independent external validation.

## 1. Introduction

Acute pulmonary embolism (PE) remains one of the most important causes of cardiovascular morbidity and mortality. Contemporary risk stratification does not rely solely on clot detection; it attempts to identify the subgroup of patients in whom right ventricular (RV) failure, systemic hypoperfusion, or early hemodynamic collapse may develop after an initially stable presentation. The 2019 European Society of Cardiology/European Respiratory Society guideline framework separates patients without overt shock or persistent hypotension into low- and intermediate-risk groups according to clinical risk scores, RV dysfunction, and myocardial injury biomarkers [1]. This approach is clinically useful, yet intermediate-risk PE remains heterogeneous: some patients remain stable with anticoagulation alone, whereas others require close monitoring, escalation of care, or rescue reperfusion. Population-level European mortality data further underline the continuing public health burden of PE [2].

The central pathophysiological event in clinically relevant PE is not merely mechanical arterial obstruction. Pulmonary vascular obstruction, hypoxic vasoconstriction, thromboinflammatory activation, and endothelial dysfunction increase RV afterload. The RV can tolerate volume loading better than abrupt pressure loading; therefore, a sudden rise in pulmonary vascular resistance may produce RV dilatation, septal shift, impaired left ventricular (LV) filling, reduced cardiac output, and eventually hemodynamic collapse. Conventional echocardiographic signs such as RV dilatation, reduced tricuspid annular plane systolic excursion (TAPSE), reduced tissue Doppler S’ velocity, septal flattening, and elevated pulmonary artery systolic pressure (PASP) are therefore important, but each captures only one component of RV adaptation.

Right ventricular-pulmonary arterial (RV-PA) coupling, most commonly approximated noninvasively by the TAPSE/PASP ratio, integrates RV longitudinal systolic shortening and pulmonary arterial afterload into a single physiological construct. A preserved ratio implies that RV contractile reserve remains proportionate to afterload, whereas a reduced ratio suggests RV-PA uncoupling. Recent studies in acute PE have shown that TAPSE/PASP decreases across risk categories, predicts early clinical deterioration or short-term outcomes, and may provide information beyond isolated TAPSE or PASP measurements [3,4,5,6]. Thus, TAPSE/PASP is attractive for intermediate-risk PE because it directly reflects the ability of the RV to adapt to the acute pulmonary vascular load.

Alongside imaging, laboratory biomarkers may capture systemic responses that are not fully visible on echocardiography. Because acute PE involves not only mechanical vascular obstruction but also endothelial activation, platelet-related thromboinflammatory activity, tissue hypoxia, and systemic organ-perfusion stress, EASIX may provide complementary information to echocardiographic RV assessment. The Endothelial Activation and Stress Index (EASIX), calculated from lactate dehydrogenase, creatinine, and platelet count, was originally described in hematologic populations as an accessible index of endothelial activation and systemic stress and has subsequently been evaluated in other disease settings [7,8]. A recent acute PE study suggested that EASIX is associated with disease severity and in-hospital prognosis [9]. However, that evidence has not clarified whether EASIX is linked to echocardiographic RV-PA uncoupling, nor whether the combination of EASIX and TAPSE/PASP improves early risk stratification specifically in hemodynamically stable intermediate-risk PE.

We therefore conducted this retrospective cohort study to evaluate a clinically applicable risk-stratification strategy in intermediate-risk acute PE. We hypothesized that elevated admission EASIX and impaired RV-PA coupling, assessed by a reduced TAPSE/PASP ratio, would be independently associated with an early adverse clinical outcome (EACO) and that their combination would provide incremental discriminatory information beyond conventional clinical variables, cardiac troponin, lactate, D-dimer, and the C-reactive protein-to-albumin ratio (CAR).

## 2. Materials and Methods

### 2.1. Study Design and Population

This was a retrospective, single-center, observational cohort study of consecutive adult patients admitted with objectively confirmed acute PE between 1 January 2020 and 10 June 2025. Acute PE was confirmed by computed tomography pulmonary angiography (CTPA) or, when CTPA was contraindicated, high-probability ventilation-perfusion imaging together with compatible clinical presentation. For patients with more than one PE-related admission during the study period, only the first eligible index admission was considered; recurrent admissions were not included in the final screening sequence. Demographic characteristics, comorbidities, vital signs, laboratory values, imaging findings, treatment details, and in-hospital outcomes were abstracted from electronic medical records, emergency department charts, laboratory databases, echocardiography reports, CTPA reports, intensive care documentation, and procedure records. The study was conducted in accordance with the Declaration of Helsinki and was approved by the Kırşehir Ahi Evran University Ethics Committee (approval date: 10 June 2025; decision no. 2025-11/127); the requirement for informed consent was waived because of the retrospective design.

### 2.2. Inclusion and Exclusion Criteria

Eligible patients were adults aged 18 years or older with symptomatic acute PE, initial hemodynamic stability, and intermediate-risk classification according to the 2019 European Society of Cardiology/European Respiratory Society (ESC/ERS) acute PE guideline framework [1]. High-risk PE was defined, in accordance with this framework, as cardiac arrest, obstructive shock, persistent systolic blood pressure < 90 mmHg, a decrease in systolic blood pressure ≥ 40 mmHg for more than 15 min not explained by arrhythmia, hypovolemia, or sepsis, or the need for vasopressor/inotropic support at presentation. Low-risk PE was defined as sPESI 0 in the absence of objective RV dysfunction and myocardial injury. Intermediate-risk PE was defined as hemodynamically stable acute PE with sPESI ≥ and/or objective evidence of RV dysfunction or myocardial injury, as recommended for post-diagnostic risk stratification in normotensive PE [1].

Clinical risk was assessed using the Pulmonary Embolism Severity Index (PESI) and the simplified Pulmonary Embolism Severity Index (sPESI), as derived from admission clinical data. PESI incorporates age, sex, comorbidities, vital signs, and oxygen saturation to estimate short-term mortality risk in acute PE, whereas sPESI is a simplified six-item version including age >80 years, history of cancer, chronic cardiopulmonary disease, heart rate ≥110 beats/min, systolic blood pressure <100 mmHg, and arterial oxygen saturation <90%. For risk categorization in the present study, sPESI was used in accordance with the ESC/ERS post-diagnostic risk stratification framework.

Intermediate-high-risk PE required both objective RV dysfunction on TTE or CTPA and positive cardiac troponin, whereas intermediate-low-risk PE included patients with only one of these features or with clinical-risk features without the combined imaging-biomarker high-risk pattern. Patients were excluded for high-risk PE at presentation, low-risk PE without intermediate-risk features, chronic thromboembolic pulmonary hypertension, known severe pulmonary hypertension unrelated to the index PE, active sepsis or clinically/radiologically evident acute pulmonary infection at presentation, hematologic platelet disorders or chemotherapy-related cytopenia expected to distort platelet counts, end-stage renal disease requiring dialysis, recurrent PE admission during the study period, missing EASIX components, missing troponin, missing arterial lactate, missing CRP or albumin, no transthoracic echocardiography performed as part of initial PE risk stratification, or technically inadequate TAPSE/PASP measurement.

### 2.3. Clinical Endpoint

The primary endpoint was EACO, a study-defined composite defined as the occurrence of at least one PE-related adverse event within the first seven days after PE diagnosis during the index hospitalization: PE-related death, cardiac arrest or cardiopulmonary resuscitation, new-onset hemodynamic decompensation, persistent systolic blood pressure <90 mmHg, requirement for vasopressor or inotropic support, unplanned ICU transfer due to PE-related respiratory or hemodynamic worsening, rescue systemic thrombolysis, catheter-directed reperfusion therapy, surgical embolectomy or mechanical thrombectomy after initial anticoagulation, invasive or noninvasive mechanical ventilation, or high-flow oxygen therapy due to PE-related respiratory worsening. The components of this composite endpoint were selected on the basis of guideline-defined hemodynamic instability and rescue reperfusion concepts and were aligned with prior intermediate-risk and normotensive PE studies evaluating early clinical deterioration [1,10,11,12,13]. Endpoint components were adjudicated from clinical notes, medication records, ICU transfer documentation, blood gas records, procedure reports, and death summaries by two investigators blinded to EASIX and TAPSE/PASP values. Disagreements were resolved by consensus with a third investigator. Treatment escalation was considered PE-related only when the indication was worsening RV failure, hypoxemia, or hemodynamic instability attributed to PE by the treating team. Events primarily attributable to alternative causes, such as sepsis, major bleeding, acute coronary syndrome, uncontrolled arrhythmia, or decompensated chronic heart failure, were not classified as PE-related EACO. Events occurring after day 7 were not counted as early outcomes in the primary analysis.

### 2.4. Laboratory Assessment and EASIX Calculation

Venous blood samples and arterial blood gas samples were obtained at emergency department presentation as part of the standard initial diagnostic and risk assessment workflow for suspected or confirmed acute PE. For patients transferred from another ward or referred from another institution, the first sample set obtained immediately at hospital presentation for the index PE episode was used. Sampling was performed before rescue reperfusion therapy, vasopressor/inotrope initiation, mechanical ventilatory escalation, or blood product administration. Venous blood for complete blood count was collected into ethylenediaminetetraacetic acid tubes and analyzed using an automated hematology analyzer (Sysmex XN-series, Sysmex Corporation, Kobe, Japan). Venous blood for routine biochemistry, including creatinine, lactate dehydrogenase (LDH), C-reactive protein (CRP), and albumin, was collected into serum-separator tubes, centrifuged according to the central laboratory protocol, and measured using an automated clinical chemistry platform (Cobas 8000 series, Roche Diagnostics, Mannheim, Germany). Venous blood for coagulation testing was collected into citrate tubes; D-dimer was measured in citrated plasma using a quantitative immunoturbidimetric assay and reported in mg/L fibrinogen equivalent units. High-sensitivity cardiac troponin I (hs-cTnI) was measured using the institutional high-sensitivity troponin I assay and was classified as positive when hs-cTnI was ≥14 pg/mL, corresponding to the assay-specific 99th percentile upper reference limit. Arterial blood gas analysis was performed on presentation blood gas samples using a point-of-care blood gas analyzer (ABL-series, Radiometer Medical, Copenhagen, Denmark), and arterial lactate was used as the primary lactate variable. Only arterial lactate was used in the primary analyses; venous lactate values, when present, were not substituted for arterial lactate in the regression models. Patients without an admission arterial lactate value were not included in the primary analytic cohort. When more than one arterial lactate measurement was available on the admission date, the earliest presentation blood gas value was used.

Admission laboratory values were therefore defined as values obtained from the first venous laboratory and arterial blood gas sample set drawn during the initial emergency department or hospital presentation for the index PE episode. Values obtained after clinical deterioration, rescue reperfusion therapy, vasopressor/inotrope treatment, mechanical ventilatory escalation, or blood product administration were not used to calculate admission biomarkers. CAR was calculated as CRP (mg/L) divided by albumin (g/L). EASIX was calculated as LDH (U/L) × creatinine (mg/dL)/platelet count (10^9^/L). Because EASIX has a right-skewed distribution, log2-EASIX was used in regression models. LDH, creatinine, and platelet count were not entered separately into multivariable models containing EASIX to avoid mathematical coupling.

### 2.5. Echocardiographic Assessment

Transthoracic echocardiography (TTE) was performed using a Toshiba Aplio Artida echocardiography system (Toshiba Medical Systems Corporation, Otawara, Japan) by certified cardiologists or trained echocardiography operators as part of the initial risk stratification after PE diagnosis. If multiple examinations were available, the earliest TTE performed after PE diagnosis and before clinical deterioration, rescue reperfusion therapy, vasopressor/inotrope initiation, or mechanical ventilatory escalation was used. Measurements were performed according to right-heart assessment recommendations [14,15]. RV-focused apical four-chamber views were used whenever available. RV basal and mid-cavity diameters were measured at end-diastole. TAPSE was obtained by M-mode alignment with the lateral tricuspid annulus and expressed in millimeters. PASP was estimated from the peak tricuspid regurgitation velocity using the modified Bernoulli equation and adding estimated right atrial pressure derived from inferior vena cava (IVC) diameter and collapsibility; PASP was recorded only when an adequate tricuspid regurgitation Doppler envelope was available. The TAPSE/PASP ratio was calculated as mm/mmHg. TAPSE/PASP was used as the primary echocardiographic index of RV-PA coupling, whereas RV/LV ratio was used as a conventional structural marker of RV dilatation in sensitivity analyses.

RV systolic velocity (S’) was measured by tissue Doppler imaging at the lateral tricuspid annulus, and RV fractional area change was calculated when image quality permitted. RV/LV ratio was measured in the apical four-chamber view at end-diastole. Septal flattening, McConnell sign, and IVC dilatation were recorded as categorical findings. In patients with atrial fibrillation or frequent ectopy, measurements were averaged across at least five representative cardiac cycles; otherwise, three cardiac cycles were averaged when feasible. Although the index TTE examination was obtained in a real-world clinical setting as part of urgent PE risk stratification, the quantitative echocardiographic measurements used for the present analysis were reviewed offline from stored images by two experienced cardiologists who were blinded to EASIX values, laboratory biomarkers, treatment escalation, and clinical outcomes. When measurements differed beyond clinically acceptable limits, images were re-evaluated jointly and a consensus value was recorded. A random subset of 90 studies was remeasured by the same and by a second observer for intra- and interobserver reproducibility using intraclass correlation coefficients.

### 2.6. CT Pulmonary Angiography Variables

CTPA was used to confirm PE and to support risk classification. Central PE location, bilateral involvement, saddle embolus, CT-derived RV/LV diameter ratio, interventricular septal bowing, and reflux of contrast into the inferior vena cava or hepatic veins were recorded when available. CT-derived RV/LV ratio was measured on axial transverse images at the level of the maximal short-axis diameters, perpendicular to the long axis of each ventricle, or abstracted from structured radiology reports when direct measurement was not feasible. CTPA variables were reviewed by investigators blinded to EASIX values and clinical outcomes. In the present study, CTPA variables were considered supportive and were placed in Supplementary Tables rather than in the final primary model to maintain a clinically practical admission model centered on readily available laboratory markers and bedside echocardiography.

### 2.7. Treatment and Clinical Follow-Up

All patients received anticoagulation and PE-directed management according to the ESC/ERS guideline recommendations [1] and the judgment of the treating team. Patients who underwent immediate reperfusion before completion of baseline laboratory and echocardiographic risk assessment were not included in the primary predictive analysis, because treatment escalation could not be temporally distinguished from the initial management decision. Routine primary reperfusion at presentation was not counted as an outcome. Only rescue reperfusion or treatment escalation occurring after initial anticoagulation because of PE-related clinical deterioration was included in the primary endpoint. For the primary endpoint, follow-up started at the time of PE diagnosis and ended at the earliest of EACO occurrence, hospital discharge, or day 7 after diagnosis. Events occurring after day 7 were not considered early outcomes for the primary analysis. Outcome ascertainment was complete through day 7 or discharge for all included patients, including those admitted near the end of the inclusion period. Pre-admission anticoagulant exposure was reviewed when it was clearly documented in medication reconciliation records. However, because therapeutic-dose versus prophylactic-dose intensity could not be reconstructed with sufficient reliability for all transferred, oncologic, postoperative, immobilized, atrial fibrillation, or prior VTE patients across the full retrospective period, pre-admission anticoagulant intensity was not used as a primary exposure variable. This limitation was considered during the interpretation of treatment-related and deterioration-related outcomes.

### 2.8. Statistical Analysis

Distributional characteristics of continuous variables were evaluated by visual inspection of histograms and Q-Q plots, skewness and kurtosis values, and the Kolmogorov–Smirnov test; distributional decisions were not based on a single test alone. Continuous variables with approximately normal distribution were summarized as mean ± standard deviation and compared using the independent-samples *t* test, whereas non-normally distributed variables were summarized as median [interquartile range] and compared using the Mann–Whitney U test. Categorical variables were presented as numbers and percentages and compared using the chi-square test or Fisher’s exact test, as appropriate. Because EASIX, lactate, D-dimer, CAR, and several echocardiographic variables were expected to show skewed distributions or monotonic but not necessarily linear relationships, Spearman rank correlation coefficients were used as the primary correlation method for analyses involving log2-EASIX and echocardiographic/laboratory variables. Pearson correlation was reserved for approximately normally distributed continuous variable pairs or for sensitivity analyses after appropriate transformation when linearity assumptions were visually supported. EASIX was log2-transformed for regression analyses to reduce skewness and to allow interpretation per doubling of the index.

Multivariable logistic regression models were constructed hierarchically. Model 1 included baseline clinical variables selected a priori: age, sex, active cancer, chronic cardiopulmonary disease, heart rate, systolic blood pressure, and oxygen saturation. Model 2 added conventional laboratory risk markers available in routine PE care: cardiac troponin positivity, lactate, D-dimer, and CAR. Model 3 added log2-transformed EASIX to Model 2, whereas Model 4 added the TAPSE/PASP ratio to Model 2. Model 5 was the final primary combined model and included both log2-EASIX and the TAPSE/PASP ratio in addition to Model 2 variables. Model 6 was a prespecified echocardiographic sensitivity model created by additionally adjusting Model 5 for RV/LV ratio, a conventional structural marker of RV dilatation, to test whether the main findings were robust beyond classical RV enlargement. Model 6 was not considered the primary model because RV/LV ratio and TAPSE/PASP partly reflect overlapping RV pathophysiology; it was used only as a robustness analysis. TAPSE and PASP were not entered separately into models containing TAPSE/PASP, and LDH, creatinine, platelet count, CRP, and albumin were not entered separately into models containing EASIX or CAR. D-dimer was log10-transformed before regression analyses because of its markedly right-skewed distribution. The modeling strategy and interpretation of predictive performance were guided by established recommendations for regression modeling and clinical prediction model development [16,17].

Model discrimination was assessed by receiver operating characteristic (ROC) curve analysis and the area under the curve (AUC). AUCs were compared using the DeLong test. Cut-offs for EASIX, TAPSE/PASP, and predicted probability of the final model were identified using the Youden index, with clinical interpretation focused on sensitivity, specificity, and negative predictive value. Calibration was evaluated using calibration plots, the Brier score, and the Hosmer-Lemeshow test. Internal validation was performed using 1000 bootstrap resamples to estimate optimism-corrected AUC and calibration slope. In addition, 10-fold cross-validation was used to estimate out-of-fold model discrimination. Patients with missing mandatory variables required for EASIX calculation, TAPSE/PASP assessment, or primary endpoint ascertainment were excluded from the primary analytic cohort. For the remaining covariates, the overall proportion of missing data was less than 5%; therefore, complete-case analysis was performed. Single mean or median imputation was not used. Multicollinearity was evaluated by variance inflation factor, with VIF > 5 considered potentially problematic. Statistical analyses were performed with IBM SPSS Statistics version 28.0 and R version 4.3.2. A two-sided *p* < 0.05 was considered statistically significant. Given the number of primary endpoint events, the final model was interpreted as a hierarchical explanatory and hypothesis-generating model rather than as a definitive clinical implementation model. Model complexity, event-per-variable considerations, optimism correction, and calibration were evaluated together, and the clinical meaning of incremental discrimination was interpreted cautiously.

### 2.9. Use of Generative Artificial Intelligence

During manuscript preparation, a generative artificial intelligence language tool was used only for language-editing, structural, and formatting assistance. No generative artificial intelligence tool was used for study design, patient selection, data extraction, endpoint adjudication, echocardiographic measurements, statistical analysis, reference selection or verification, generation of tables or figures, or interpretation of patient-level data. All manuscript content, citations, source-dependent statements, analyses, tables, and figures were reviewed, verified, and approved by the authors, who take full responsibility for the integrity and accuracy of the work.

## 3. Results

**Figure 1:**
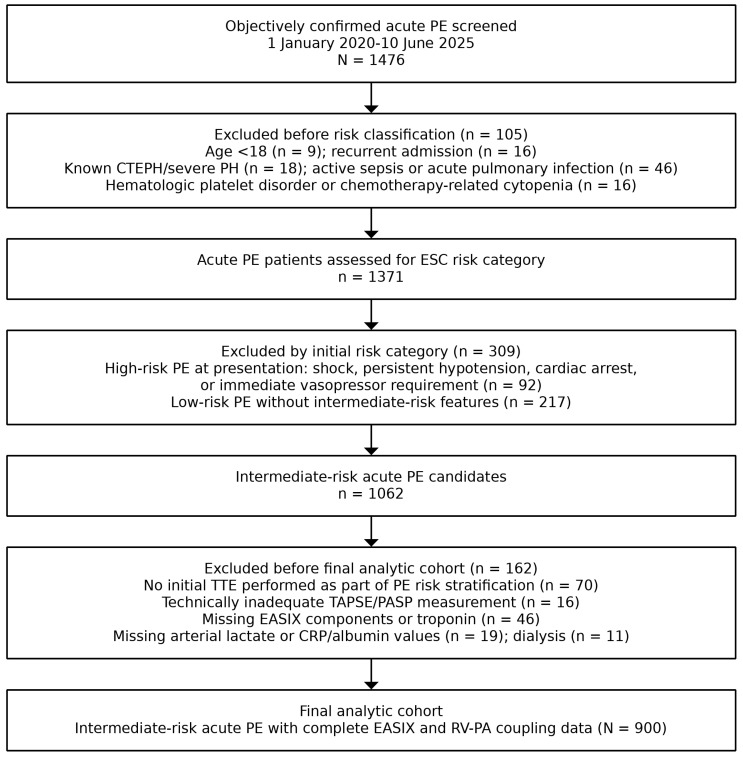
Patient inclusion flowchart.

Among 1476 screened patients with objectively confirmed acute PE between 1 January 2020 and 10 June 2025, 900 hemodynamically stable intermediate-risk patients with complete admission EASIX and TAPSE/PASP data comprised the final analytic cohort (Figure 1). Of the 162 patients excluded before the final analytic cohort, 70 had no initial transthoracic echocardiography performed as part of PE risk stratification and 16 had a technically inadequate TAPSE/PASP measurement; the remaining exclusions were due to missing laboratory variables or dialysis, as shown in Figure 1. The median age was 67 [58–76] years, 424 patients (47.1%) were male, 140 (15.6%) had active cancer, and 383 (42.6%) were classified as intermediate-high risk.

EACO occurred in 110 patients (12.2%) within the first 7 days after PE diagnosis. ICU transfer due to PE-related respiratory or hemodynamic worsening occurred in 73 patients (8.1%), rescue reperfusion therapy in 37 (4.1%), vasopressor/inotrope requirement in 40 (4.4%), ventilatory support in 30 (3.3%), and PE-related death in 14 (1.6%). Endpoint components were not mutually exclusive.

Baseline clinical and laboratory characteristics are summarized in Table 1. Patients with EACO were older and had a higher heart rate, lower systolic blood pressure, lower mean arterial pressure, lower oxygen saturation, a higher respiratory rate, a higher sPESI score, and a higher proportion of intermediate-high-risk classification than those without events. Troponin positivity was more frequent in patients with EACO than in those without EACO (68.2% vs. 42.2%, *p* < 0.001). Median lactate (2.3 [1.8–2.9] vs. 1.6 [1.2–2.0] mmol/L, *p* < 0.001), D-dimer (6.3 [4.3–10.6] vs. 4.7 [3.1–7.0] mg/L FEU, *p* < 0.001), CAR (1.24 [0.83–1.93] vs. 0.89 [0.58–1.42], *p* < 0.001), EASIX (2.28 [1.52–3.27] vs. 1.27 [0.86–2.00], *p* < 0.001), and log2-EASIX (1.19 [0.60–1.71] vs. 0.34 [−0.21–1.00], *p* < 0.001) were higher in the EACO group.

**Figure 2:**
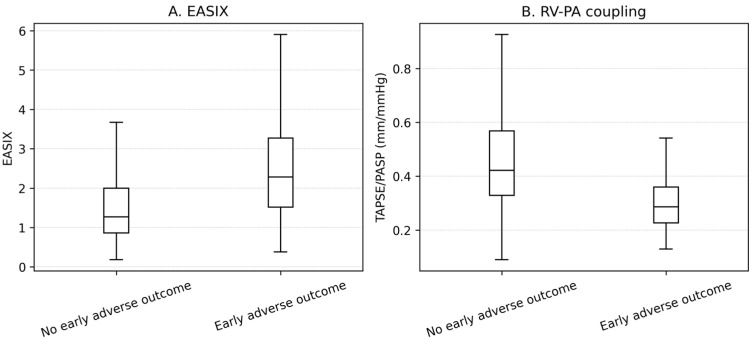
Distribution of admission EASIX and TAPSE/PASP ratio according to EACO. EASIX, Endothelial Activation and Stress Index; TAPSE, tricuspid annular plane systolic excursion; PASP, pulmonary artery systolic pressure; RV-PA, right ventricular-pulmonary arterial.

Echocardiographic findings are presented in Table 2. Compared with patients without EACO, patients with EACO had higher RV/LV diameter ratio (1.26 [1.10–1.41] vs. 1.05 [0.88–1.20], *p* < 0.001), lower TAPSE (14.8 [13.4–17.4] vs. 18.4 [15.7–21.4] mm, *p* < 0.001), higher PASP (52 [44–65] vs. 43 [34–53] mmHg, *p* < 0.001), lower RV S’ velocity (8.9 [7.5–10.1] vs. 10.6 [9.1–12.3] cm/s, *p* < 0.001), lower RV fractional area change (31% [25–36] vs. 37% [31–42], *p* < 0.001), and lower TAPSE/PASP ratio (0.29 [0.23–0.36] vs. 0.42 [0.33–0.57] mm/mmHg, *p* < 0.001). Composite echocardiographic RVD was present in 110 patients with EACO and 697 patients without EACO (100.0% vs. 88.2%, *p* < 0.001). Septal flattening/D-shaped LV (53.6% vs. 39.7%, *p* = 0.008) and IVC dilatation/reduced collapse (49.1% vs. 30.8%, *p* < 0.001) were also more frequent in the EACO group. Right atrial area was larger in patients with EACO (23.7 [20.5–27.7] vs. 19.9 [16.6–23.1] cm^2^, *p* < 0.001). In univariable analysis, right atrial area was associated with EACO (OR 1.16 per cm^2^, 95% CI 1.12–1.21, *p* < 0.001; Supplementary Table S1). The distribution of admission EASIX and TAPSE/PASP ratio according to the occurrence of EACO is shown in Figure 2.

Several comorbidities differed between groups. Heart failure, chronic obstructive pulmonary disease, and prior venous thromboembolism were more frequent in patients with EACO than in those without EACO, as shown in Table 1. Chronic cardiopulmonary disease was included among the clinical covariates in the hierarchical regression models.

**Figure 3:**
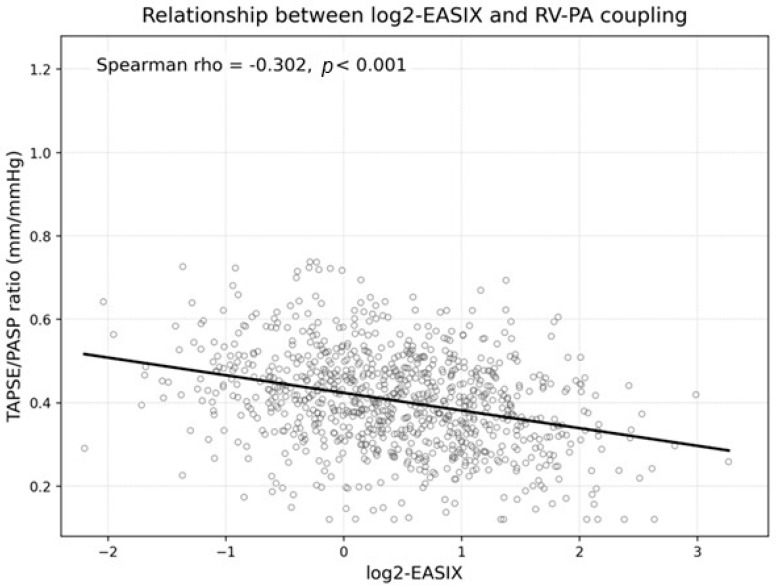
Relationship between log2-EASIX and TAPSE/PASP ratio. EASIX, Endothelial Activation and Stress Index; TAPSE, tricuspid annular plane systolic excursion; PASP, pulmonary artery systolic pressure; RV-PA, right ventricular-pulmonary arterial.

In correlation analyses, log2-EASIX showed a significant inverse association with TAPSE/PASP ratio (Spearman rho = −0.302, *p* < 0.001), TAPSE (rho = −0.204, *p* < 0.001), RV S’ velocity (rho = −0.187, *p* < 0.001), and RV fractional area change (rho = −0.180, *p* < 0.001). Positive correlations were observed between log2-EASIX and PASP (rho = 0.278, *p* < 0.001), RV/LV ratio (rho = 0.280, *p* < 0.001), lactate (rho = 0.195, *p* < 0.001), CAR (rho = 0.192, *p* < 0.001), and D-dimer (rho = 0.148, *p* < 0.001) (Table 3; Figure 3).

In multivariable analyses, both log2-EASIX and TAPSE/PASP remained independently associated with EACO in the final combined model after adjustment for clinical variables, troponin positivity, lactate, D-dimer, and CAR (Table 4). In Model 5, log2-EASIX was associated with higher odds of EACO per doubling of EASIX (OR 1.55, 95% CI 1.14–2.10, *p* = 0.005). Each 0.1-unit decrease in TAPSE/PASP was also associated with higher odds of EACO (OR 1.36, 95% CI 1.10–1.68, *p* = 0.004). In the echocardiographic sensitivity model additionally adjusted for the RV/LV ratio, log2-EASIX (OR 1.49, 95% CI 1.09–2.05, *p* = 0.013) and TAPSE/PASP (OR 1.30 per 0.1-unit decrease, 95% CI 1.04–1.62, *p* = 0.021) remained associated with EACO.

**Figure 4:**
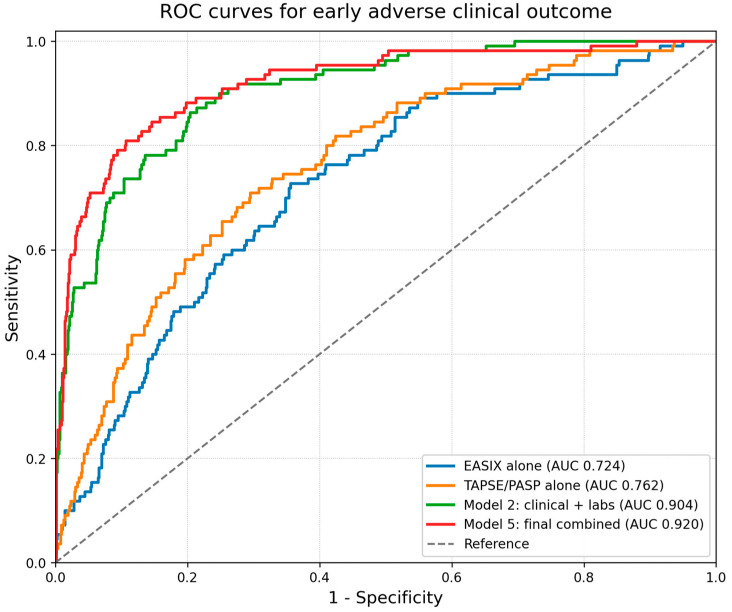
ROC curves comparing EASIX, TAPSE/PASP, the clinical-laboratory model, and the final combined model. ROC, receiver operating characteristic; EASIX, Endothelial Activation and Stress Index; TAPSE, tricuspid annular plane systolic excursion; PASP, pulmonary artery systolic pressure.

For prediction of EACO, EASIX alone yielded an AUC of 0.724 (95% CI 0.674–0.775), and the TAPSE/PASP alone yielded an AUC of 0.762 (95% CI 0.713–0.811). The clinical model had an AUC of 0.794 (95% CI 0.752–0.836), which increased to 0.904 (95% CI 0.870–0.938) after conventional laboratory variables were added. Adding EASIX to the clinical-laboratory model increased the AUC to 0.911 (95% CI 0.878–0.944; DeLong *p* = 0.048 vs. Model 2), adding TAPSE/PASP increased it to 0.918 (95% CI 0.887–0.949; DeLong *p* = 0.010 vs. Model 2), and the final combined model including both variables achieved an AUC of 0.920 (95% CI 0.889–0.951; DeLong *p* = 0.006 vs. Model 2) (Table 5; Figure 4). The absolute AUC increase from Model 2 to the final combined model was 0.016.

**Figure 5:**
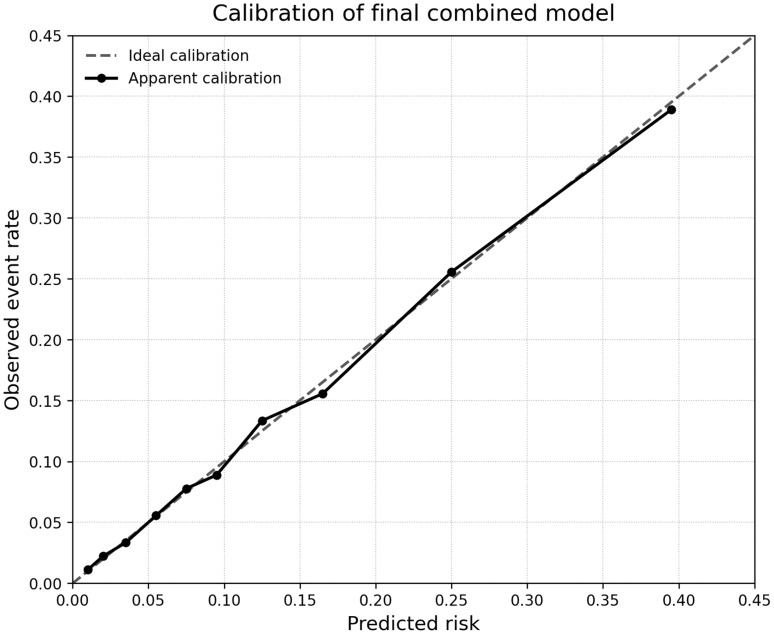
Calibration plot of the final combined model for EACO. EACO, early adverse clinical outcome; calibration slope and intercept reflect internal model calibration.

The ROC-derived cut-off for EASIX was >1.65, with 71.8% sensitivity and 64.4% specificity. The ROC-derived cut-off for TAPSE/PASP was ≤0.37, with 79.1% sensitivity and 63.0% specificity. The final model predicted-probability cut-off of >0.19 provided 79.1% sensitivity, 88.9% specificity, 49.7% positive predictive value, and 96.8% negative predictive value. Bootstrap optimism-corrected AUC was 0.910, 10-fold cross-validated AUC was 0.904, and the Brier score was 0.084. Calibration was acceptable visually and by Hosmer-Lemeshow testing (*p* = 0.621), with a calibration slope of 0.96 and intercept of −0.01 (Figure 5).

## 4. Discussion

In this retrospective cohort of 900 hemodynamically stable intermediate-risk acute PE patients, the principal findings were as follows. First, admission EASIX was significantly higher in patients who developed EACO. Second, impaired RV-PA coupling, expressed by a reduced TAPSE/PASP ratio, was strongly associated with early deterioration and treatment escalation. Third, log2-EASIX correlated inversely with the TAPSE/PASP and with several RV functional parameters, suggesting a biologically plausible association between systemic endothelial-organ stress and RV afterload maladaptation. Fourth, EASIX and TAPSE/PASP remained independently associated with EACO after adjustment for clinical variables, troponin, lactate, D-dimer, and CAR. Finally, the combined model showed only a modest absolute improvement in discrimination beyond conventional clinical-laboratory assessment but remained stable after bootstrap and cross-validation. These findings support a hypothesis-generating integrated risk phenotype rather than an immediately deployable treatment algorithm.

The clinical relevance of this approach lies in the heterogeneity of intermediate-risk PE. The ESC framework appropriately emphasizes hemodynamic status, clinical risk, RV dysfunction, and biomarkers [1]. However, patients within this category do not follow a uniform clinical course. Some have a substantial clot burden but preserved RV reserve, whereas others are normotensive at presentation despite limited hemodynamic reserve and subsequent need for escalation of care. Leidi et al. [18] highlighted that risk stratification remains a cornerstone of PE management, while also noting that more refined tools are still needed for the intermediate-risk group. Our strategy addresses this gap by combining an echocardiographic marker of RV adaptation with a simple laboratory marker of systemic endothelial and organ stress.

The rationale for focusing on RV-PA coupling also has a strong foundation in earlier PE literature, even though older studies usually used the broader terminology of RV dysfunction rather than ventricular-arterial coupling. Grifoni et al. [19] showed that normotensive patients with acute PE and echocardiographic RV dysfunction had a substantially higher risk of PE-related shock and in-hospital death than those without RV dysfunction, supporting the concept of latent hemodynamic vulnerability despite preserved systemic blood pressure. Kucher et al. [20] subsequently demonstrated in a large registry cohort that RV hypokinesis predicted 30-day mortality among PE patients presenting with a systolic blood pressure of 90 mmHg or higher. Bova et al. [21] further refined this concept by combining clinical presentation, RV dysfunction, and myocardial injury to identify normotensive PE patients at higher short-term risk. Together, these studies established the clinical importance of RV maladaptation in apparently stable PE; contemporary TAPSE/PASP-based approaches extend this paradigm by relating RV contractile performance to pulmonary arterial afterload rather than assessing RV dilatation or hypokinesis alone. Pruszczyk et al. [22] also demonstrated the prognostic value of echocardiographic assessment in normotensive acute PE, further supporting the clinical relevance of conventional RV functional evaluation in this population.

The TAPSE/PASP ratio is attractive because it captures two essential components of acute PE pathophysiology: RV systolic shortening and pulmonary arterial afterload. TAPSE alone can be deceptively preserved or reduced depending on loading conditions, imaging alignment, and the regional nature of RV contraction. PASP alone quantifies afterload but does not show whether the RV can tolerate that afterload. The ratio therefore approximates RV-PA coupling, a concept increasingly recognized in pulmonary vascular disease and acute PE. Lyhne et al. [3] reported that TAPSE/PASP has prognostic value in acute PE and decreases with increasing PE severity. Zuin et al. [4] showed prognostic value for early clinical deterioration and short-term mortality in intermediate-high-risk PE, while Mostafa et al. [5] further supported the ability of echocardiographic RV-PA coupling assessment to predict adverse outcomes. Yuriditsky et al. [6] extended this concept to invasive hemodynamic correlates in acute PE patients undergoing mechanical thrombectomy, linking RV-PA uncoupling with a reduced cardiac index and normotensive shock. Cimini et al. [23] also reinforced the prognostic importance of echocardiographic RV dysfunction parameters in acute PE. Our study builds on these reports by embedding TAPSE/PASP into a multimarker model rather than treating it as a stand-alone echocardiographic variable.

The EASIX component of the model provides a complementary biological dimension. Luft et al. [7] introduced EASIX in patients with acute graft-versus-host disease as a readily available index associated with endothelial complications and outcomes, and Song et al. [8] subsequently supported its prognostic relevance in multiple myeloma. Its components have plausible relevance in acute PE: LDH may reflect cellular injury, tissue hypoxia, and endothelial damage; creatinine may reflect renal hypoperfusion, venous congestion, or systemic hemodynamic stress; and platelet count may reflect platelet activation, consumption, and thromboinflammatory engagement. The biological plausibility of EASIX in acute PE is further supported by the thromboinflammatory nature of venous thromboembolism. Imiela et al. [24] emphasized that acute PE involves not only pulmonary arterial obstruction but also platelet activation, endothelial dysfunction, and inflammatory cascade activation. Imiela et al. [25] further described acute PE as a multifactorial thromboinflammatory process involving coagulation, platelets, endothelium, immune cells, oxidative stress, and related mediators. Therefore, EASIX may not merely reflect a nonspecific laboratory abnormality; rather, it may integrate downstream consequences of acute PE, including tissue hypoxia, renal-perfusion impairment, platelet-related thromboinflammatory activation, and endothelial-organ stress. Karayigit et al. [9] recently reported that EASIX was associated with disease severity and prognosis in acute PE. That study is important because it establishes EASIX as a plausible PE marker. However, it primarily positioned EASIX as a broad severity and mortality marker in an all-comer acute PE population. The current model addresses a narrower and clinically more difficult question: among initially stable intermediate-risk PE patients, does EASIX add information to the echocardiographic assessment of RV-PA coupling and routine admission markers?

The inverse relationship between log2-EASIX and TAPSE/PASP is pathophysiologically plausible. Acute PE can provoke abrupt pulmonary vascular obstruction, hypoxic vasoconstriction, endothelial perturbation, platelet-leukocyte activation, and RV pressure overload. When RV contractile reserve becomes insufficient relative to afterload, systemic consequences may follow: reduced forward output, hepatic and renal congestion, impaired renal perfusion, tissue hypoxia, and biochemical evidence of cellular stress. In this setting, the TAPSE/PASP represents the mechanical expression of RV maladaptation, whereas EASIX represents the biochemical expression of endothelial-organ stress. Their combination may therefore identify a high-risk phenotype that neither imaging nor laboratory assessment alone can fully characterize.

Combining EASIX with TAPSE/PASP is useful because the two variables provide complementary rather than redundant information. The TAPSE/PASP describes the mechanical interaction between RV systolic performance and pulmonary arterial afterload, whereas EASIX reflects the systemic biochemical consequences of endothelial activation, cellular stress, renal-perfusion impairment, and platelet-related thromboinflammatory activity. A patient with impaired TAPSE/PASP but low EASIX may predominantly express a mechanical RV afterload phenotype, whereas a patient with elevated EASIX but relatively preserved TAPSE/PASP may express systemic endothelial-organ stress without overt RV-PA uncoupling. The coexistence of both abnormalities may define a more advanced high-risk phenotype in which RV maladaptation and systemic stress amplification occur simultaneously. Accordingly, the combined model should be viewed not as a replacement for guideline-based intermediate-risk classification, but as a tool to identify the higher-risk phenotype hidden within this broad category before overt hypotension, shock, or rescue reperfusion becomes necessary.

The inclusion of troponin, lactate, D-dimer, and CAR in the laboratory model was intentional. Troponin reflects RV myocardial injury and is integrated into contemporary risk stratification [1]. Lactate has particular value in normotensive PE because it may detect occult tissue hypoperfusion before overt hypotension. Vanni et al. [10] showed that elevated plasma lactate identifies normotensive PE patients at increased risk of short-term PE-related complications, and Ebner et al. [11] demonstrated that venous lactate improves the prediction of adverse in-hospital outcomes. D-dimer is primarily diagnostic but may provide a rough representation of fibrin turnover and thrombotic activity. The CAR adds inflammatory and negative acute-phase information; Ozcan et al. [26] and Artac et al. [27] reported its prognostic relevance in acute PE. By adjusting for these variables, the present model tests whether EASIX and TAPSE/PASP offer information beyond established and readily available markers. In addition, Najarro et al. [28] linked C-reactive protein with right ventricular dysfunction and mortality in acute symptomatic PE, supporting the broader inflammatory-risk context in which CAR was included.

The echocardiographic sensitivity model provides additional context for interpreting the primary findings. TAPSE/PASP reflects functional RV-PA coupling, whereas the RV/LV ratio is a conventional structural marker of RV dilatation. Because these parameters capture related but not identical aspects of right-heart involvement in acute PE, adjustment for the RV/LV ratio allowed us to examine whether the associations of EASIX and TAPSE/PASP were maintained beyond overt RV enlargement. The persistence of both associations in this sensitivity model suggests that the combined biochemical and functional phenotype was not explained solely by conventional RV dilatation. Qualitative pressure-overload signs, such as septal flattening, were retained in descriptive echocardiographic analyses but were not forced into the primary model to reduce overadjustment and collinearity among closely related right-heart variables.

A practical advantage of this model is that it avoids dependence on NT-proBNP, which is unavailable in many real-world centers. This is relevant for hospitals where troponin, D-dimer, lactate, CRP, albumin, LDH, creatinine, and platelet count are routinely obtained but natriuretic peptides are not. The model therefore reflects a realistic emergency and cardiology workflow: blood gas lactate and routine laboratory tests provide rapid biochemical information, while a focused TTE provides TAPSE, PASP, and conventional RV assessment. The final model is not intended to replace guideline-based risk stratification; rather, it may help identify intermediate-risk patients who need closer monitoring, ICU-level observation, PERT discussion, or early reassessment.

The question of RV strain is relevant. Right ventricular global or free-wall longitudinal strain may detect subtle dysfunction and has shown prognostic value in some intermediate-risk PE studies. Eguchi et al. [29], for example, reported the additive value of RV global longitudinal strain with the RV/LV ratio for 30-day mortality in intermediate-risk PE. Tzourtzos et al. [30] summarized a growing body of strain literature but also emphasized heterogeneity across studies. In acute PE, strain analysis requires adequate RV-focused images, vendor/software compatibility, post-processing expertise, and time. For a real-world early risk model, especially in dyspneic or unstable patients, routine TAPSE/PASP is more practical. Therefore, RV strain would be best treated as an optional exploratory substudy in patients with adequate archived images rather than as a core requirement of the primary model.

The main contribution of this study over previous work is its integrated phenotype approach. Existing PE studies have evaluated clinical scores, troponin, natriuretic peptides, lactate, inflammatory markers, the CT RV/LV ratio, conventional echocardiography, and TAPSE/PASP separately. The recent EASIX-PE literature suggests that EASIX may have prognostic value, but does not fully connect it to RV-PA coupling. Conversely, TAPSE/PASP studies demonstrate the importance of RV adaptation to afterload, but do not evaluate the systemic endothelial-organ stress response. Thus, whereas previous acute PE work positioned EASIX mainly as a broad severity and mortality marker, the present study positions EASIX as the biochemical counterpart of RV-PA uncoupling and early clinical deterioration within the clinically ambiguous intermediate-risk subgroup. By combining EASIX and TAPSE/PASP with troponin, lactate, D-dimer, and CAR, the results support a clinically plausible pathway in which pulmonary vascular obstruction and endotheliopathy are linked to RV-PA uncoupling, systemic stress, and early deterioration.

From a clinical perspective, the potential value of this approach is not to replace guideline-based risk stratification or to directly trigger reperfusion therapy, but to help characterize the heterogeneity within the intermediate-risk PE category. If validated externally, a combined EASIX and TAPSE/PASP-based phenotype may help identify patients who warrant closer observation, early reassessment, or a multidisciplinary discussion, while patients with a low predicted risk may represent a more stable subgroup. However, any clinical use of such a model must be integrated with hemodynamic status, oxygenation, bleeding risk, imaging findings, and physician judgment.

Chronic cardiopulmonary comorbidity may modify the relationship between acute embolic obstruction, RV-PA coupling, and early clinical deterioration. Patients with COPD, heart failure, or prior VTE may have limited RV reserve, a higher baseline pulmonary vascular load, impaired gas exchange, or pre-existing cardiopulmonary remodeling. In this setting, even a moderate acute increase in pulmonary vascular resistance may result in a disproportionate reduction in RV-PA coupling. Therefore, the associations observed in the present study should not be interpreted solely as a reflection of acute embolic burden, but rather as the combined effect of acute PE, pre-existing cardiopulmonary reserve, and systemic endothelial-organ stress. This interpretation is also consistent with the inclusion of chronic cardiopulmonary disease in the sPESI and with the broader ESC/ERS framework for post-diagnostic risk stratification [1].

Right atrial enlargement also deserves comment. In the present cohort, the right atrial area was significantly larger among patients with EACO and was associated with EACO in univariable analysis. A larger right atrium may reflect pressure-volume burden, impaired right-sided compliance, venous congestion, and reduced reserve even when individual RV systolic parameters are only moderately abnormal. Therefore, the right atrial area may represent an additional supportive echocardiographic marker of risk. However, right atrial fractional area change was not routinely available in the retrospective echocardiographic archive, and right atrial measurements were not incorporated into the primary model to avoid overfitting and collinearity with other right-heart variables.

Despite these interpretive cautions, this study also has several methodological strengths: consecutive patient inclusion, explicit intermediate-risk definitions, the exclusion of confounders that distort EASIX components, a structured hierarchical modeling strategy, the avoidance of mathematical coupling, robust sensitivity modeling, calibration analysis, and internal validation. These design decisions address common reviewer concerns in retrospective biomarker studies, including unplanned model selection, collinearity, and uncertain clinical applicability; nevertheless, they do not eliminate the need for external validation.

## 5. Limitations

This study has several limitations. First, the retrospective single-center design may introduce selection bias, residual confounding, and center-specific management effects despite multivariable adjustment and internal validation. The study period extended over more than five years, during which institutional thresholds for ICU transfer, rescue reperfusion, oxygen escalation, and timing or quality of echocardiography may have evolved. Therefore, the proposed model may not be directly transportable to centers with different PE response workflows, echocardiography availability, or escalation protocols.

Second, EACO was a study-defined composite endpoint. Although its components were selected according to guideline-defined hemodynamic instability, rescue reperfusion concepts, and prior normotensive or intermediate-risk PE deterioration studies, the individual components differ in clinical severity. PE-related death and cardiac arrest are not clinically equivalent to ICU transfer, high-flow oxygen use, or noninvasive ventilatory support. Therefore, the composite should be interpreted together with its components, and the possibility that associations are partly influenced by less severe or practice-dependent components cannot be excluded.

Third, EASIX should be interpreted as a nonspecific marker of endothelial activation, organ stress, renal-perfusion impairment, and platelet-related thromboinflammatory burden rather than as a PE-specific mechanistic biomarker. LDH, creatinine, and platelet count may be affected by renal dysfunction, hemolysis, liver disease, malignancy, occult infection, inflammatory disorders, and hematologic conditions. Although major confounders were excluded or adjusted for, residual confounding cannot be eliminated.

Fourth, TAPSE/PASP is a practical non-invasive surrogate of RV-PA coupling, but it does not directly measure invasive ventricular-arterial coupling. Its accuracy depends on adequate acoustic windows, reliable TAPSE acquisition, measurable tricuspid regurgitation Doppler envelopes, and appropriate estimation of right atrial pressure. Patients with unavailable baseline echocardiography or technically inadequate TAPSE/PASP measurement were excluded, which may introduce selection bias if these patients differed systematically from the analytic cohort. Figure 1 distinguishes patients excluded because no initial echocardiography was available (*n* = 70) from those with a technically inadequate TAPSE/PASP measurement (*n* = 16). However, the retrospective dataset did not contain sufficiently complete harmonized baseline information for all excluded patients to support a reliable included-versus-excluded comparison; therefore, the possibility of selection bias cannot be completely excluded. This limitation should be addressed in future prospective validation studies with mandatory standardized echocardiographic acquisition.

Fifth, NT-proBNP was not included because it was not routinely available during the study period. While this may increase the real-world applicability of the model in centers without routine natriuretic peptide testing, it limits direct comparison with biomarker-based guideline algorithms. RV free-wall longitudinal strain was also not incorporated into the primary model because it requires adequate RV-focused image quality, dedicated software, and offline post-processing, which may reduce feasibility in emergency PE assessment. Right atrial fractional area change was likewise not routinely available in the retrospective echocardiographic archive.

Sixth, pre-admission anticoagulant exposure may also influence the clinical phenotype at presentation. Therapeutic-dose anticoagulation for atrial fibrillation or prior VTE, and prophylactic anticoagulation in postoperative, immobilized, or oncologic patients, may modify thrombotic burden, embolic recurrence, and the early deterioration risk. In this retrospective cohort, however, anticoagulant type, dose intensity, adherence, timing of last dose, and perioperative interruption could not be reconstructed with sufficient reliability for the entire study period. We therefore avoided presenting an imprecise subgroup analysis and acknowledge this as a limitation.

Seventh, this study evaluated admission values only; serial changes in EASIX, lactate, troponin, and TAPSE/PASP were not assessed. Therefore, the model does not capture dynamic improvement or deterioration after anticoagulation or reperfusion therapy. In addition, long-term outcomes such as recurrent venous thromboembolism, post-PE functional limitation, RV recovery, chronic thromboembolic pulmonary hypertension, and long-term mortality were not evaluated.

Eighth, the modest AUC increment should be interpreted carefully. Although the final model showed a statistically detectable increase in AUC compared with the clinical-laboratory model, the absolute difference was small. Therefore, the potential clinical value of the combined model should not be inferred from AUC alone. Its possible utility lies in combining bedside feasibility, high negative predictive value, calibration, and a physiologically interpretable phenotype, all of which require external validation before clinical implementation.

Finally, the number of EACO events limits the certainty of a multivariable model containing multiple clinical, laboratory, and echocardiographic predictors. Although the modeling strategy was hierarchical and internally validated, internal validation cannot fully remove overfitting, optimism, or center-specific model performance. The final model should therefore be considered an explanatory model and a basis for future validation rather than a definitive clinical prediction rule. The derived EASIX, TAPSE/PASP, and predicted-risk thresholds should not be used as definitive treatment triggers before validation in independent, preferably multicenter prospective cohorts.

## 6. Conclusions

In this retrospective cohort study of intermediate-risk acute PE, admission EASIX and TAPSE/PASP may provide complementary information for early risk stratification. Elevated EASIX reflected systemic endothelial-organ stress, while a reduced TAPSE/PASP identified impaired RV-PA coupling. Their combination was associated with EACO beyond clinical variables, troponin, lactate, D-dimer, and CAR, although the incremental AUC gain over the clinical-laboratory model was modest. These findings should be considered as preliminary and require external validation before the model or its thresholds are used for clinical decision-making or treatment escalation.

## Figures and Tables

**Table 1:** Baseline clinical and laboratory characteristics.

Variable	Overall (*n* = 900)	No EACO (*n* = 790)	EACO (*n* = 110)	*p* Value
Age, years	67 [58–76]	66 [58–75]	71 [61–80]	<0.001
Male sex	424 (47.1)	371 (47.0)	53 (48.2)	0.890
Active cancer	140 (15.6)	114 (14.4)	26 (23.6)	0.018
Chronic cardiopulmonary disease	198 (22.0)	171 (21.6)	27 (24.5)	0.572
Heart failure	122 (13.6)	97 (12.3)	25 (22.7)	0.004
Chronic obstructive pulmonary disease	151 (16.8)	119 (15.1)	32 (29.1)	<0.001
Prior VTE	150 (16.7)	116 (14.7)	34 (30.9)	<0.001
Recent surgery or trauma	150 (16.7)	130 (16.5)	20 (18.2)	0.750
Immobilization within previous 4 weeks	188 (20.9)	168 (21.3)	20 (18.2)	0.535
Syncope at presentation	60 (6.7)	51 (6.5)	9 (8.2)	0.634
Heart rate, beats/min	95 [82–108]	95 [81–107]	101 [89–114]	<0.001
Systolic blood pressure, mmHg	123 [111–135]	124 [113–136]	115 [102–126]	<0.001
Mean arterial pressure, mmHg	89 [80–97]	89 [81–98]	82 [73–92]	<0.001
Oxygen saturation, %	92.9 [90.4–95.8]	93.3 [90.7–96.0]	90.6 [87.7–92.7]	<0.001
Respiratory rate, breaths/min	22 [19–25]	22 [19–24]	24 [22–27]	<0.001
sPESI score	1 [1–2]	1 [1–1]	2 [1–2]	<0.001
Intermediate-high risk category	383 (42.6)	313 (39.6)	70 (63.6)	<0.001
Hemoglobin, g/dL	13.1 [11.9–14.3]	13.1 [11.9–14.3]	12.7 [11.4–13.8]	0.013
White blood cell count, 10^9^/L	9.4 [7.2–11.8]	9.3 [7.2–11.6]	10.0 [7.7–12.7]	0.024
Neutrophil-to-lymphocyte ratio	4.3 [3.5–5.5]	4.3 [3.5–5.5]	4.4 [3.6–6.0]	0.519
Platelet count, 10^9^/L	235 [184–286]	237 [186–290]	217 [161–265]	0.004
LDH, U/L	311 [244–380]	301 [238–367]	399 [310–478]	<0.001
Creatinine, mg/dL	1.03 [0.85–1.26]	1.01 [0.83–1.22]	1.21 [0.97–1.46]	<0.001
eGFR, mL/min/1.73 m^2^	99 [79–122]	100 [80–124]	83 [61–106]	<0.001
Sodium, mmol/L	138 [136–140]	138 [136–140]	138 [135–140]	0.284
High-sensitivity troponin positive	408 (45.3)	333 (42.2)	75 (68.2)	<0.001
Lactate, mmol/L	1.6 [1.2–2.1]	1.6 [1.2–2.0]	2.3 [1.8–2.9]	<0.001
D-dimer, mg/L FEU	4.8 [3.3–7.3]	4.7 [3.1–7.0]	6.3 [4.3–10.6]	<0.001
C-reactive protein, mg/L	35.2 [23.1–54.1]	33.4 [22.2–51.8]	45.4 [31.2–68.2]	<0.001
Albumin, g/L	37.9 [34.8–40.8]	38.0 [34.8–41.0]	36.8 [34.2–40.0]	0.023
CAR	0.93 [0.60–1.48]	0.89 [0.58–1.42]	1.24 [0.83–1.93]	<0.001
EASIX	1.34 [0.90–2.18]	1.27 [0.86–2.00]	2.28 [1.52–3.27]	<0.001
log2-EASIX	0.42 [−0.15–1.12]	0.34 [−0.21–1.00]	1.19 [0.60–1.71]	<0.001

Values are median [interquartile range] or *n* (%). sPESI, simplified Pulmonary Embolism Severity Index; VTE, venous thromboembolism; EASIX, Endothelial Activation and Stress Index = LDH × creatinine/platelet count; LDH, lactate dehydrogenase; CAR, C-reactive protein-to-albumin ratio; FEU, fibrinogen equivalent units; EACO, early adverse clinical outcome.

**Table 2:** Echocardiographic characteristics at admission.

Variable	Overall (*n* = 900)	No EACO (*n* = 790)	EACO (*n* = 110)	*p* Value
LVEF, %	59 [55–63]	59 [55–63]	57 [52–62]	0.004
RV basal diameter, mm	41 [36–45]	40 [36–45]	45 [41–50]	<0.001
RV mid-cavity diameter, mm	34 [30–39]	34 [30–39]	38 [32–44]	<0.001
RV/LV diameter ratio	1.07 [0.91–1.24]	1.05 [0.88–1.20]	1.26 [1.10–1.41]	<0.001
TAPSE, mm	18.1 [15.1–20.9]	18.4 [15.7–21.4]	14.8 [13.4–17.4]	<0.001
PASP, mmHg	44 [35–54]	43 [34–53]	52 [44–65]	<0.001
RV S’ velocity, cm/s	10.4 [8.9–12.0]	10.6 [9.1–12.3]	8.9 [7.5–10.1]	<0.001
RV fractional area change, %	36 [30–42]	37 [31–42]	31 [25–36]	<0.001
TAPSE/PASP ratio, mm/mmHg	0.40 [0.31–0.54]	0.42 [0.33–0.57]	0.29 [0.23–0.36]	<0.001
Right atrial area, cm^2^	20.3 [16.8–23.8]	19.9 [16.6–23.1]	23.7 [20.5–27.7]	<0.001
IVC dilatation/reduced collapse	297 (33.0)	243 (30.8)	54 (49.1)	<0.001
Septal flattening/D-shaped LV	373 (41.4)	314 (39.7)	59 (53.6)	0.008
McConnell sign	177 (19.7)	143 (18.1)	34 (30.9)	0.002
Composite echocardiographic RVD	807 (89.7)	697 (88.2)	110 (100.0)	<0.001

Values are median [interquartile range] or *n* (%). PASP, pulmonary artery systolic pressure; TAPSE, tricuspid annular plane systolic excursion; RV, right ventricle; LV, left ventricle; LVEF, left ventricular ejection fraction; IVC, inferior vena cava; RVD, right ventricular dysfunction; EACO, early adverse clinical outcome.

**Table 3:** Spearman correlations between log2-EASIX and selected echocardiographic/laboratory variables.

Variable	Spearman r	*p* Value
TAPSE/PASP ratio	−0.302	<0.001
TAPSE	−0.204	<0.001
PASP	0.278	<0.001
RV/LV ratio	0.280	<0.001
RV basal diameter	0.166	<0.001
RV S’ velocity	−0.187	<0.001
RV FAC	−0.180	<0.001
Lactate	0.195	<0.001
CAR	0.192	<0.001
D-dimer	0.148	<0.001

Negative correlations indicate lower values of the echocardiographic variable with increasing EASIX.

**Table 4:** Hierarchical logistic regression models for EACO in intermediate-risk acute PE.

Variable	Unadjusted	Model 1 Clinical	Model 2 Clinical + Lab	Model 3 Model 2 + EASIX	Model 4 Model 2 + TAPSE/PASP	Model 5 Final Combined	Model 6 Sensitivity: Model 5 + RV/LV Ratio
Age, per 10 years	1.28 (1.12–1.47) *p* = 0.001	1.17 (1.01–1.36) *p* = 0.041	1.14 (0.98–1.33) *p* = 0.086	1.13 (0.97–1.32) *p* = 0.118	1.12 (0.96–1.31) *p* = 0.143	1.10 (0.94–1.30) *p* = 0.236	1.09 (0.93–1.29) *p* = 0.281
Male sex	1.09 (0.72–1.64) *p* = 0.689	1.05 (0.68–1.61) *p* = 0.826	1.03 (0.66–1.60) *p* = 0.903	1.02 (0.65–1.58) *p* = 0.945	1.01 (0.65–1.58) *p* = 0.958	0.99 (0.63–1.56) *p* = 0.972	0.98 (0.62–1.55) *p* = 0.944
Active cancer	2.06 (1.29–3.28) *p* = 0.002	1.72 (1.04–2.84) *p* = 0.034	1.61 (0.96–2.71) *p* = 0.072	1.54 (0.91–2.60) *p* = 0.109	1.51 (0.89–2.56) *p* = 0.126	1.42 (0.83–2.43) *p* = 0.198	1.40 (0.81–2.42) *p* = 0.226
Chronic cardiopulmonary disease	1.78 (1.14–2.77) *p* = 0.011	1.43 (0.89–2.30) *p* = 0.141	1.34 (0.82–2.20) *p* = 0.242	1.31 (0.79–2.17) *p* = 0.294	1.27 (0.76–2.13) *p* = 0.359	1.22 (0.72–2.07) *p* = 0.458	1.20 (0.70–2.05) *p* = 0.505
Heart rate, per 10 bpm	1.31 (1.17–1.47) *p* < 0.001	1.22 (1.08–1.39) *p* = 0.002	1.17 (1.02–1.34) *p* = 0.023	1.15 (1.00–1.32) *p* = 0.047	1.13 (0.98–1.30) *p* = 0.095	1.10 (0.95–1.28) *p* = 0.204	1.09 (0.94–1.27) *p* = 0.247
Systolic BP, per 10-mmHg decrease	1.39 (1.21–1.60) *p* < 0.001	1.28 (1.10–1.50) *p* = 0.002	1.23 (1.04–1.45) *p* = 0.014	1.20 (1.02–1.42) *p* = 0.031	1.18 (0.99–1.40) *p* = 0.061	1.14 (0.95–1.37) *p* = 0.157	1.13 (0.94–1.36) *p* = 0.187
Oxygen saturation, per 5% decrease	1.52 (1.27–1.82) *p* < 0.001	1.32 (1.08–1.61) *p* = 0.007	1.25 (1.01–1.54) *p* = 0.040	1.21 (0.98–1.50) *p* = 0.079	1.19 (0.96–1.48) *p* = 0.115	1.14 (0.91–1.43) *p* = 0.246	1.13 (0.90–1.42) *p* = 0.289
Troponin positivity	2.83 (1.88–4.26) *p* < 0.001	-	2.02 (1.27–3.20) *p* = 0.003	1.88 (1.17–3.01) *p* = 0.009	1.74 (1.08–2.81) *p* = 0.023	1.55 (0.94–2.57) *p* = 0.087	1.51 (0.91–2.51) *p* = 0.111
Lactate, per 1 mmol/L	1.82 (1.48–2.24) *p* < 0.001	-	1.50 (1.18–1.90) *p* = 0.001	1.40 (1.10–1.79) *p* = 0.007	1.35 (1.05–1.73) *p* = 0.018	1.27 (0.98–1.64) *p* = 0.071	1.25 (0.96–1.62) *p* = 0.094
D-dimer, log10-transformed	1.46 (1.16–1.84) *p* = 0.001	-	1.21 (0.93–1.58) *p* = 0.157	1.15 (0.88–1.52) *p* = 0.305	1.16 (0.88–1.54) *p* = 0.285	1.08 (0.81–1.45) *p* = 0.595	1.07 (0.80–1.44) *p* = 0.641
CAR, per 1 unit	1.24 (1.14–1.35) *p* < 0.001	-	1.15 (1.04–1.27) *p* = 0.006	1.11 (1.00–1.24) *p* = 0.049	1.12 (1.01–1.25) *p* = 0.034	1.08 (0.96–1.21) *p* = 0.195	1.07 (0.95–1.20) *p* = 0.260
log2-EASIX	2.31 (1.82–2.94) *p* < 0.001	-	-	1.82 (1.39–2.39) *p* < 0.001	-	1.55 (1.14–2.10) *p* = 0.005	1.49 (1.09–2.05) *p* = 0.013
TAPSE/PASP, per 0.1-unit decrease	1.71 (1.45–2.03) *p* < 0.001	-	-	-	1.49 (1.23–1.81) *p* < 0.001	1.36 (1.10–1.68) *p* = 0.004	1.30 (1.04–1.62) *p* = 0.021
RV/LV diameter ratio, per 0.1 increase	1.29 (1.14–1.46) *p* < 0.001	-	-	-	-	-	1.16 (1.01–1.35) *p* = 0.041
C-statistic/AUC	-	0.794	0.904	0.911	0.918	0.920	0.929
Optimism-corrected AUC	-	0.780	0.889	0.895	0.902	0.910	0.914
Hosmer-Lemeshow *p* value	-	0.372	0.448	0.514	0.567	0.621	0.604

Values are presented as odds ratio (95% confidence interval), *p* value, unless otherwise indicated. Unadjusted estimates were derived from separate univariable logistic regression models. Model 5 is interpreted as a prespecified explanatory and hypothesis-generating model. EACO, early adverse clinical outcome; PE, pulmonary embolism; EASIX, Endothelial Activation and Stress Index; TAPSE, tricuspid annular plane systolic excursion; PASP, pulmonary artery systolic pressure; RV, right ventricle; LV, left ventricle; CAR, C-reactive protein-to-albumin ratio.

**Table 5:** Discrimination, cut-offs, and internal validation for prediction of EACO.

Predictor/Model	AUC (95% CI)	Cut-Off	Sensitivity, %	Specificity, %	DeLong *p* vs. Model 2
EASIX alone	0.724 (0.674–0.775)	>1.65	71.8	64.4	<0.001
TAPSE/PASP alone	0.762 (0.713–0.811)	≤0.37	79.1	63.0	0.001
Model 1: clinical	0.794 (0.752–0.836)	Predicted probability	-	-	<0.001
Model 2: clinical + labs	0.904 (0.870–0.938)	Predicted probability	-	-	Reference
Model 3: Model 2 + EASIX	0.911 (0.878–0.944)	Predicted probability	-	-	0.048
Model 4: Model 2 + TAPSE/PASP	0.918 (0.887–0.949)	Predicted probability	-	-	0.010
Model 5: final combined	0.920 (0.889–0.951)	>0.19	79.1	88.9	0.006
Model 6: sensitivity + RV/LV ratio	0.929 (0.900–0.958)	Predicted probability	-	-	0.004

The final model cut-off refers to predicted probability using the Youden index. Model 5 also showed a 10-fold cross-validated AUC of 0.904, bootstrap optimism-corrected AUC of 0.910, Brier score of 0.084, calibration slope of 0.96, calibration intercept of −0.01, and Hosmer-Lemeshow *p* = 0.621. AUC, area under the ROC curve; CI, confidence interval.

## Data Availability

The datasets analyzed during the current study are available from the corresponding author on reasonable request, subject to institutional and ethical restrictions.

## Supplementary Materials

The following supporting information can be downloaded at: https://www.mdpi.com/article/10.3390/jcm15145675/s1, Figure S1: Early adverse outcome rates across combined EASIX/RV-PA coupling phenotypes; Figure S2: Decision curve analysis comparing the clinical-laboratory model and final combined model; Table S1: Univariate logistic regression analyses for early adverse clinical outcomes; Table S2: Outcomes according to combined EASIX/RV-PA coupling phenotype; Table S3: Supportive CTPA variables.
