# Supplementary material for: Admission Endothelial Activation and Stress Index and Echocardiographic RV-PA Coupling for Early Risk Stratification in Intermediate-Risk Acute Pulmonary Embolism
Source: J Clin Med. 2026 Jul 20;15(14):5675. doi: 10.3390/jcm15145675 (PMC13413401; doi:10.3390/jcm15145675)
Supplement: Supplementary file 1 [file jcm-15-05675-s001.zip › jcm-4412468-supplementary.pdf]

Supplementary Table S1. Univariate logistic regression analyses for early adverse clinical outcomes.

Variable	Unadjusted OR	95% CI	p value
Age, years	1.03	1.02-1.05	<0.001
Male sex	1.05	0.70-1.57	0.810
Active cancer	1.84	1.13-2.97	0.014
Chronic cardiopulmonary disease	1.18	0.74-1.88	0.492
Heart failure	2.10	1.28-3.44	0.003
Chronic obstructive pulmonary disease	2.31	1.47-3.65	<0.001
Prior VTE	2.60	1.66-4.08	<0.001
Recent surgery or trauma	1.13	0.67-1.90	0.649
Immobilization within previous 4 weeks	0.82	0.49-1.38	0.457
Syncope at presentation	1.29	0.62-2.70	0.498
Heart rate, beats/min	1.02	1.01-1.03	<0.001
Systolic blood pressure, mmHg	0.96	0.95-0.98	<0.001
Mean arterial pressure, mmHg	0.96	0.94-0.98	<0.001
Oxygen saturation, %	0.81	0.77-0.86	<0.001
Respiratory rate, breaths/min	1.16	1.10-1.22	<0.001
sPESI score	2.71	2.09-3.50	<0.001
Intermediate-high risk category	2.67	1.76-4.03	<0.001
Hemoglobin, g/dL	0.88	0.78-0.98	0.025
White blood cell count, 10^9/L	1.07	1.01-1.14	0.019
Neutrophil-to-lymphocyte ratio	1.06	0.92-1.21	0.434
Platelet count, 10^9/L	1.00	0.99-1.00	0.002
LDH, U/L	1.01	1.01-1.01	<0.001
Creatinine, mg/dL	6.19	3.35-11.44	<0.001
eGFR, mL/min/1.73 m^2	0.98	0.97-0.98	<0.001
Sodium, mmol/L	0.96	0.91-1.02	0.236
High-sensitivity troponin positive	2.94	1.92-4.50	<0.001
Lactate, mmol/L	4.14	3.06-5.60	<0.001
D-dimer, mg/L FEU	1.12	1.07-1.16	<0.001
C-reactive protein, mg/L	1.01	1.01-1.02	<0.001
Albumin, g/L	0.94	0.90-0.99	0.010
CRP/albumin ratio	1.67	1.40-2.01	<0.001
EASIX	1.53	1.35-1.75	<0.001
log2-EASIX	2.40	1.91-3.03	<0.001
LVEF, %	0.95	0.92-0.98	0.002
RV basal diameter, mm	1.13	1.09-1.17	<0.001
RV mid-cavity diameter, mm	1.10	1.07-1.14	<0.001
RV/LV diameter ratio, per 0.1 increase	1.48	1.35-1.63	<0.001
TAPSE, mm	0.82	0.78-0.87	<0.001
PASP, mmHg	1.06	1.04-1.07	<0.001
RV S' velocity, cm/s	0.71	0.65-0.78	<0.001
RV fractional area change, %	0.91	0.89-0.94	<0.001
TAPSE/PASP ratio, per 0.1-unit decrease	1.71	1.45-2.03	<0.001
Right atrial area, cm^2	1.16	1.12-1.21	<0.001
IVC dilatation/reduced collapse	2.17	1.45-3.25	<0.001
Septal flattening/D-shaped LV	1.75	1.17-2.62	0.006
McConnell sign	2.02	1.30-3.15	0.002

Supplementary Table S2. Outcomes according to combined EASIX/RV-PA coupling phenotype.

Phenotype	n	Early adverse outcome	ICU transfer	Rescue reperfusion	PE-related death
Low EASIX / preserved coupling	332	11 (3.3)	6 (1.8)	2 (0.6)	1 (0.3)
High EASIX / preserved coupling	242	20 (8.3)	11 (4.5)	7 (2.9)	1 (0.4)
Low EASIX / impaired coupling	118	11 (9.3)	7 (5.9)	4 (3.4)	1 (0.8)
High EASIX / impaired coupling	208	68 (32.7)	49 (23.6)	24 (11.5)	11 (5.3)

High EASIX was defined as above the cohort median; impaired coupling was defined as TAPSE/PASP <=0.35 for this phenotype analysis.

Supplementary Table S3. Supportive CTPA variables.

Variable	Overall	No early adverse outcome	Early adverse outcome	p value
Central or saddle PE	314 (34.9)	248 (31.4)	66 (60.0)	<0.001
Bilateral PE	642 (71.3)	544 (68.9)	98 (89.1)	<0.001
CT RV/LV ratio	1.12 [0.97-1.31]	1.09 [0.95-1.26]	1.35 [1.19-1.54]	<0.001
Septal bowing on CTPA	248 (27.6)	186 (23.5)	62 (56.4)	<0.001
IVC/hepatic vein contrast reflux	284 (31.6)	221 (28.0)	63 (57.3)	<0.001

These CTPA variables were included for descriptive enrichment and sensitivity planning but were not part of the primary admission model.

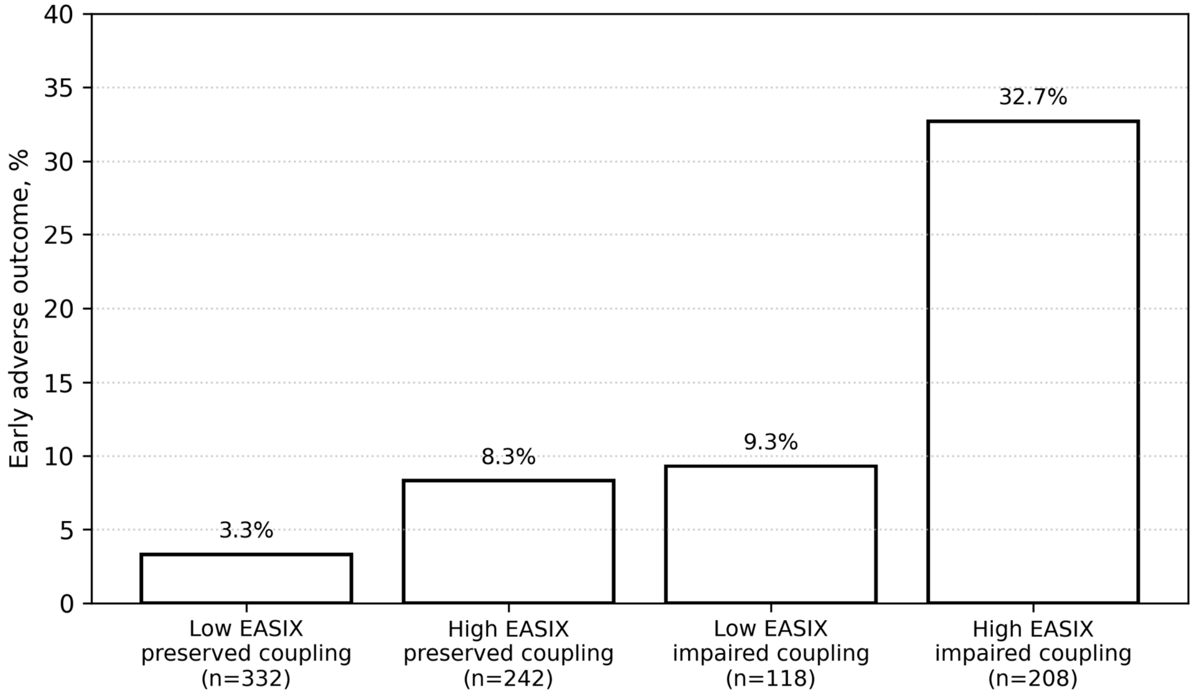

Supplementary Figure S1. Early adverse outcome rates across combined EASIX/RV-PA coupling phenotypes.

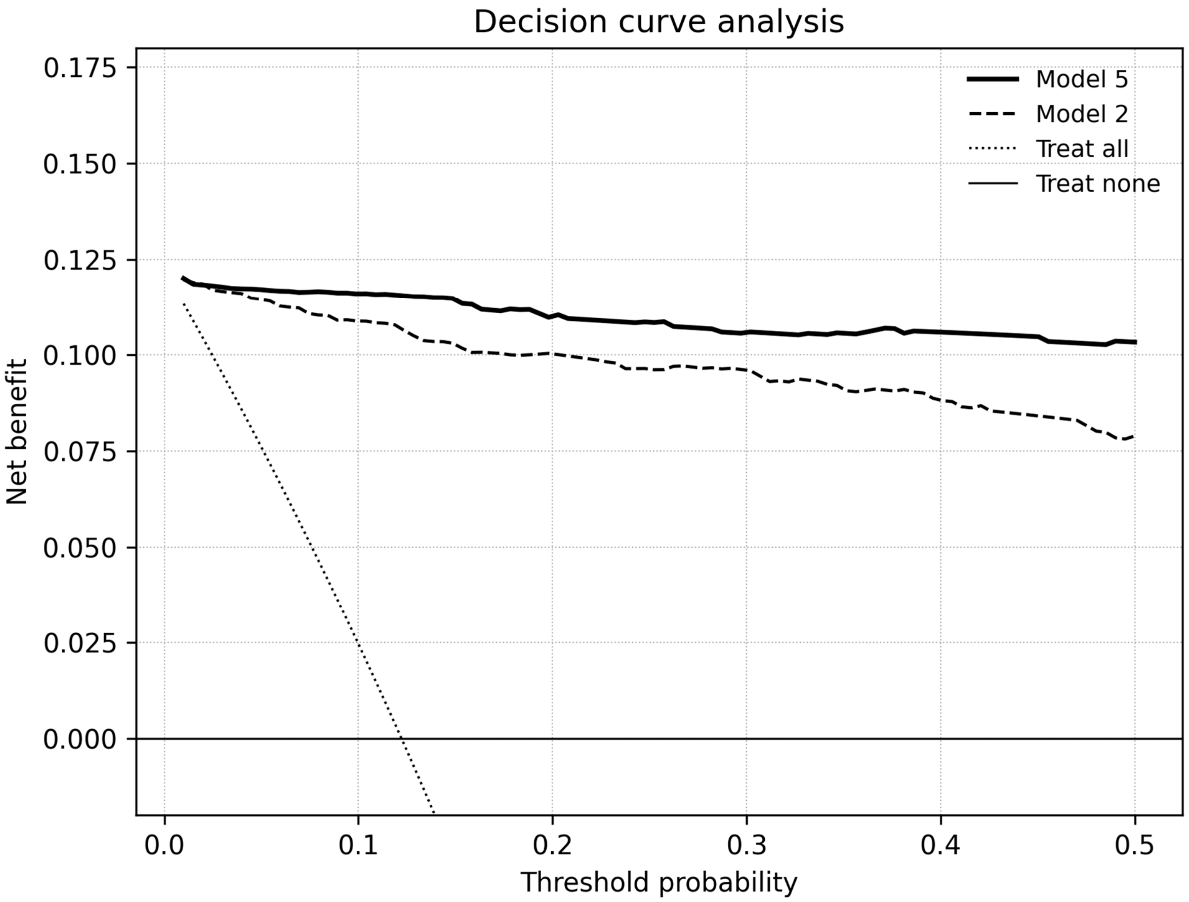

Supplementary Figure S2. Decision curve analysis comparing the clinical-laboratory model and final combined model.
